# Suicide and Youth: Risk Factors

**DOI:** 10.3389/fpsyt.2018.00540

**Published:** 2018-10-30

**Authors:** Johan Bilsen

**Affiliations:** ^1^Mental Health and Wellbeing Research Group, Department of Public Health, Vrije Universiteit Brussel, Brussels, Belgium; ^2^Public Mental Health Section, European Public Health Association (EUPHA), Utrecht, Netherlands

**Keywords:** public mental health, suicide, youth, risk factors, prevention

## Abstract

Suicide occurs more often in older than in younger people, but is still one of the leading causes of death in late childhood and adolescence worldwide. This not only results in a direct loss of many young lives, but also has disruptive psychosocial and adverse socio-economic effects. From the perspective of public mental health, suicide among young people is a main issue to address. Therefore we need good insight in the risk factors contributing to suicidal behavior in youth. This mini review gives a short overview of the most important risk factors for late school-age children and adolescents, as established by scientific research in this domain. Key risk factors found were: mental disorders, previous suicide attempts, specific personality characteristics, genetic loading and family processes in combination with triggering psychosocial stressors, exposure to inspiring models and availability of means of committing suicide. Further unraveling and knowledge of the complex interplay of these factors is highly relevant with regard to the development of effective prevention strategy plans for youth suicide.

## Introduction

Suicide is defined as a fatal self-injurious act with some evidence of intent to die (1). Worldwide, more than 800,000 people die due to suicide each year. It is estimated that about 1.5 million people will die due to suicide by the year 2020. The suicide mortality rate in 2015 was 10.7 per 100,000, which means about one death every 20 s. Suicide accounts for 1.4% of all deaths, and is the 15th leading cause of death globally (2, 3). Many more men than women die by suicide. The male-to-female ratio varies between 4 to 1 (Europe and Americas) and 1.5 to 1 (Eastern Meditarranean and Western Pacific region), and is highest in richer countries (4). These suicide figures are probably still an underestimation of the real cases. Registering a suicide is a complicated process, often involving judicial authorities. Suicide deaths may not be recognized or may be misclassified as an accident or another cause of death. Sometimes suicide is not acknowledged or reported, due to its sensitive nature and the taboo that still surrounds it (5). Suicide attempts, i.e. non-fatal suicidal behavior, are much more frequent, and are estimated to be about 10–20 times more frequent than actual suicide. The estimated global annual prevalence of self-reported suicide attempts is approximately 3 per 1,000 adults. About 2.5% of the population makes at least one suicide attempt during their lifetime (6, 7).

Suicide rates vary substantially between regions. About 80% of all suicides occur in low and middle-income countries (8). Suicide mortality rates vary from 15.6 per 100,000 inhabitants in South-East Asia to 5.6 per 100,000 in the Eastern Mediterranean region. Europe has an average suicide mortality rate of 14.1 per 100,000, way above the global average of 10.7 per 100,000. There is wide variation between the European countries, from about 3.3 per 100,000 in Azerbaijan to tenfold that figure, 32.7 per 100,000, in Lithuania. In general, Eastern and Central European countries have the highest suicide mortality rate, Western and Northern European countries are situated around the European average, and the Mediterranean countries have the lowest rates (3).

Suicide affects all age groups in the population, but worldwide, rates clearly rise with increasing age. In almost all regions in the world, the highest rates are found among the oldest people aged 80+ (60.1 per 100,000 men and 27.8 per 100,000 women), 70–79 years (42.2 and 18.7 respectively), and 60–69 years (28.2 and 12.4 respectively). In younger people, these figures are much lower: 15.3 and 11.2 per 100,000 males and females aged between 15–29 years and 0.9 and 1.0 per 100,000 for the age category of 5–14 years. In Europe the same tendency is found, with rates decreasing from 53.2 and 14.0 per 100,000 men and women aged 80+ to respectively 19.9 and 4.2 per 100,000 for the age category 15–29 years and 1.0 and 0.4 for the age category of 5–14 years (9). Notwithstanding the lower suicide rates among the younger age groups, suicide is the second leading cause of death among 15–29 year olds globally (8). Also in Europe, where youth suicide rates are tending to decrease, suicide is ranked as the second most frequent cause of death in the 10–19 year age group. It is even the most frequent cause of death among females aged 15–19 years (6.15 per 100,000). Suicide deaths account for about one fifth of all deaths among European older adolescents and young adults together (15–29 years), representing about 24,000 deaths each year (10, 11). In comparison, suicide is not even in the top ten most frequent causes of death in the older age groups. These facts, together with the finding that overall these figures have not tended to decline clearly and steadily over recent decades, have caused growing concern among scientists and policy makers. There is also an increasing awareness in the general population about the tremendous negative consequences of youth suicidality, not only because of the direct loss of many young lives but also in the disruptive psychosocial and adverse socio-economic effects on a large societal scale. From the perspective of public mental health, suicide among young people is one of the main issues to address through effective preventive measures. Therefore it is important to gain as much insight as possible in the risk factors contributing to suicidal behavior in youth. In what follows, this mini review gives a short overview of the most important risk factors as established by scientific research in this domain.

## Risk factors for suicide in youth

The definition of youth in terms of strict age ranges is rather arbitrary and varies by country and over time (12). Suicide under the age of 5 is hard to find. Most literature (including this mini review) on youth suicide refers to school-age children (7–12 years) and adolescents (13–20 years). These young people are by nature vulnerable to mental health problems, especially during the years of adolescence (13). This period in life is characterized by movement, changes and transitions from one state into another, in several domains at the same time. Young people have to make decisions about important concrete directions in life, for example school, living situation, peer group etc. They must also address new challenges with regard to building their own identity, developing self-esteem, acquiring increasing independence and responsibility, building new intimate relationships, etc. In the meantime they are subject to ongoing, changing psychological and physical processes themselves. And besides that they are often confronted with high expectations, sometimes too high, from significant relatives and peers. Such situations inevitably provoke a certain degree of helplessness, insecurity, stress and a sense of losing control (14). To address these challenges and successfully cope with these emotions, young people must have access to significant supporting resources such as a stable living situation, intimate friendships, a structural framework and economic resources. Risk factors can be seen as factors that undermine this support or hinder access to these resources, while protective factors strengthen and protect these resources, or serve as a buffer against risk factors.

In recent decades, several population-based psychological autopsy studies of suicides have been conducted, involving interviews with key informants and examination of records, as well as follow-up studies of people who have attempted suicide, revealing important information about the risk factors for youth suicide (15). Everyone agrees that numerous factors can contribute to suicide, and that ultimately each suicide is caused by a highly unique, dynamic and complex interplay of genetic, biological, psychological and social factors (16). Nevertheless, it is possible to identify different types of factors that are clearly associated with an increased risk of youth suicide, so this is highly relevant with regard to prevention.

### Mental disorders

Most studies agree that suicide is closely linked to mental disorders (17, 18). About 90% of people who commit suicide have suffered from at least one mental disorder (19). Mental disorders are found to contribute between 47 and 74% of suicide risk. Affective disorder is the disorder most frequently found in this context. Criteria for depression were found in 50–65% of suicide cases, more often among females than males. Substance abuse, and more specifically alcohol misuse, is also strongly associated with suicide risk, especially in older adolescents and males. Among 30–40% of people who die by suicide had personality disorders, such as borderline or antisocial personality disorder. Suicide is often the cause of death in young people with eating disorders, in particular anorexia nervosa, as well as in people with schizophrenia, although schizophrenia as such accounts for very few of all youth suicides (17, 20). Finally, associations have also been found between suicide and anxiety disorders, but it is difficult to assess the influence of mood and substance abuse disorders that are also often present in these cases. In general, the comorbidity of mental disorders substantially increases suicide risk. Especially important here is the high prevalence of comorbidity between affective and substance abuse disorders.

### Previous suicide attempts

Many studies find a strong link between previous suicide attempts, or a history of self-harm, and suicide (21). About 25–33% of all cases of suicide were preceded by an earlier suicide attempt, a phenomenon that was more prevalent among boys than girls. Research has shown that boys with a previous suicide attempt have a 30-fold increase in suicide risk compared to boys who have not attempted suicide. Girls with previous suicide attempts have a threefold increase in suicide risk. In prospective studies, it was found that 1–6% of people attempting suicide die by suicide in the first year. The risk of suicide is found to be related mainly to the self-inflicting act as such, and less to the degree of suicidal intention of that act.

### Personality characteristics

Suicide is associated with impulsivity (22). Although we know that a suicidal process can take weeks, months or even years, the fatal transition from suicidal ideation and suicide attempts to an actual completed suicide often occurs suddenly, unexpectedly and impulsively, especially among adolescents. Difficulties in managing the various, often strong and mixed emotions and mood fluctuations accompanying the confrontation with new and ever-changing challenges in different domains is another risk factor for youth suicide, probably partly influenced by bio-neurological factors. Young people who committed suicide were also found to have had poorer problem-solving skills than their peers. Their behavior was characterized by a rather passive attitude, waiting for someone else to solve the problem for them, for simple problems as well as for more complex interpersonal problems. Some researchers indicate defects of memory in this context, with few detailed memories of effective solutions in the past (22). Others link it to the rigid thinking process often found in these young people. In this way of thinking, also called “dichotomy thinking,” people experience events and express their experiences as totally “black” or “white,” totally good or totally bad, with little space for nuance and gradation. This also accounts for their self-image. This inability in problem solving and mood regulation often causes insecurity, low self-efficacy and self-esteem, but it can also lead to anger and aggressive behavior, emotional crisis and suicidal crisis, especially in combination with perfectionist personalities (16, 23).

### Family factors

One of the most important sources of support with addressing the many challenges of youth is the family context in which young people live or have grown up. Several risk factors concerning family structure and processes have been linked to suicide behavior in numerous studies (24). It is estimated that in 50% of youth suicide cases, family factors are involved. One important factor is a history of mental disorders among direct family members themselves, especially depression and substance abuse (25). It is not clear whether these disorders directly influence the suicidal behavior of the child, or rather do so indirectly, through mental disorders evoked in the child as a result of this family context. Researchers also found an augmented presence of suicidal behavior among family members of young people who have committed suicide (17). There has been a lot of discussion about the mechanisms behind this finding. There may certainly be a kind of imitation behavior in the child, but adoption studies have reported a greater concordance of suicidal behavior with biological relatives than adoptive relatives, which points more toward a genetic explanation (26). The latter is also in line with the fact that sometimes the suicidal behavior of the parents occurred in the past, without the child's knowledge. Probably genetics and imitation both play a role (27). Poor communication within the family is also found in many cases of suicide, not only with the child or about the child's problems, but in general between family members. Direct conflicts with parents have a great impact, but so do the absence of communication and neglect of communication needs (25, 28). Furthermore, violence at home often seems to be found in the background history of young suicide cases, not only specifically against the child, but more as a way of dealing with problems between family members. Parental divorce as such is only weakly associated with suicide of the children involved, and this association is probably confounded by the practical, financial and socio-economic implications of living in a single-parent family or relational background factors related to the divorce (29).

### Specific life events-traits

Risk factors directly linked to specific important life events can be of course very diverse, but some types of event stressors are found to be more often associated with suicide in youth than others. In the context of addressing new challenges, building their own identity and establishing self-confidence, most young people attach great importance to being part of peer groups, developing new intimate relationships, establishing confidence and security. Therefore, it is not very surprising that interpersonal losses such as relationship break-ups, the death of friends and peer rejection may have a great impact in youth, and are found in one fifth of youth suicide cases (30). Other important suicide-related stressors are linked to the important domains of school and family (31). School problems and academic stress was found in 14% of suicide cases. Youngsters who are “drifting,” neither attending school nor doing a job, have substantially more risk of suicide, due to a lack of structure and predictability. Often suicide occurs after a period of absence from school, especially for young people under the age of 15. Acute conflicts with parental figures precede 40% of suicide cases (23, 32). Other concrete stressful events associated with suicide were bullying, cyber bullying, mental and physical/sexual abuse and disciplinary trouble, e.g., with police, which is more common among suicide cases with substance abuse disorders (33, 34).

### Contagion-imitation

Younger people are more suggestible and thus more prone to contagion by the behavior of others than older people are (19). Several researchers suggest using the term imitation rather than contagion. Contagion suggests a kind of infectious disease, precluding the “infected” persons' ability to act and decide for themselves. Imitation refers to learning by modeling, the acquisition of new patterns of behavior though observation of the model's behavior. Imitation of suicide behavior by youngsters can be evoked at a macro level (e.g., by mass media reports), but is also likely to be caused by direct contact in their living environment (e.g., peer groups, friends, school environment). Research shows that imitating effects may depend on a number of factors (35). Firstly, the characteristics of the model are important. In general, there are stronger imitating effects when there are similarities between the young person and the model (e.g., in age, gender, mood status, or background situation), when there is a strong bond between them, or when the model is someone they admire (e.g., celebrities). Secondly, it is important whether and to what extent the model's behavior is reinforced. The more this behavior is condoned, regarded as positive, understandable, sometimes even admirable and brave, the more young people are likely to imitate it. Thirdly, the frequency and manner of presentation of the model's behavior is important, e.g., the size and number of headlines, number of repetitions, real story or fiction. Research has shown a dose-effect relation. Sometimes this imitation behavior can take on large dimensions, known as suicide clusters, which are a chain of actual suicides, usually among adolescents, in a discrete area and period of time (36).

### Availability of means

People thinking about suicide are usually ambivalent about that decision. The transition from suicidal ideation to actual suicide often occurs impulsively as a reaction to acute psychosocial stressors, especially among young people. Availability of means of committing suicide can be crucial for that transition in that moment and that specific situation, and the method chosen may also determine the lethality of the action. Sometimes it is even linked to national patterns found in suicide methods. In line with this, children usually commit suicide by hanging, jumping from a high place or running into traffic, and poisoning with prescription drugs they have saved up. Adolescents use more varied methods: besides hanging and poisoning, young men especially also use firearms. Some studies have shown that restricting the physical availability of means of committing suicide can be important in prevention strategies (2, 37). Cognitive availability can also play an important role in youth suicide, especially in the suicidal process leading to suicide, e.g., sensationalized media reporting or detailed internet information about means and methods of committing suicide (38–40).

## Conclusion

Youth suicide constitutes a major public mental health problem. Young people and especially adolescents are by nature a vulnerable group for mental health problems. While suicide is relatively rare in children, its prevalence increases significantly throughout adolescence. And although youth suicide rates are slightly decreasing within the European region, it still ranks as a leading cause of death among the young worldwide and, as such, it is responsible for a substantial number of premature deaths and a huge amount of pointless suffering and societal loss. Each suicide is the result of a complex dynamic and unique interplay between numerous contributing factors, and individual efforts to predict and prevent suicide tend to fail. On the other hand, our knowledge of risk factors is increasing substantially. Mental disorders, previous suicide attempts, specific personality characteristics, genetic loading and family processes in combination with triggering psychosocial stressors, exposure to inspiring models and availability of means of committing suicide are key risk factors in youth suicide. The only way forward is to reduce these risk factors and strengthen protective factors as much as possible by providing integrated and multi-sector (primary, secondary and tertiary) prevention initiatives. Key prevention strategies can be population-based (e.g., mental health promotion, education, awareness by campaigns on mental resilience, careful media coverage, limited access to means of committing suicide) as well as targeting high-risk subgroups (e.g., specific school-based programmes, educating gatekeepers in different domains, providing crisis hotlines and online help, detecting and coaching dysfunctional families) or even focusing on individuals identified as suicidal (e.g., improving mental health treatment, follow-up after suicide attempts and strategies for coping with stress and grief) (41). To increase successful attempts to address youth suicide in the future, further unraveling of the complex suicide process must be accompanied by sustained and substantial efforts in scientifically underpinning and (re)evaluating ongoing and new prevention strategy plans, and this is largely a matter of policy priorities and commitment.

## Author contributions

The author confirms being the sole contributor of this work and has approved it for publication.

### Conflict of interest statement

The author declares that the research was conducted in the absence of any commercial or financial relationships that could be construed as a potential conflict of interest.
